# Update on the Genetics of Idiopathic Hypogonadotropic Hypogonadism

**DOI:** 10.4274/jcrpe.2017.S010

**Published:** 2017-12-30

**Authors:** A. Kemal Topaloğlu

**Affiliations:** 1 University of Mississippi Medical Center, Department of Pediatrics, Division of Pediatric Endocrinology and Department of Neurobiology and Anatomical Sciences, Jackson, Mississippi, USA; 2 Çukurova University Faculty of Medicine, Department of Pediatrics, Division of Pediatric Endocrinology, Adana, Turkey

**Keywords:** Hypogonadism, hypogonadotropic, delayed puberty, genetics, etiology

## Abstract

Traditionally, idiopathic hypogonadotropic hypogonadism (IHH) is divided into two major categories: Kallmann syndrome (KS) and normosmic IHH (nIHH). To date, inactivating variants in more than 50 genes have been reported to cause IHH. These mutations are estimated to account for up to 50% of all apparently hereditary cases. Identification of further causative gene mutations is expected to be more feasible with the increasing use of whole exome/genome sequencing. Presence of more than one IHH-associated mutant gene in a given patient/pedigree (oligogenic inheritance) is seen in 10-20% of all IHH cases. It is now well established that about 10-20% of IHH cases recover from IHH either spontaneously or after receiving some sex steroid replacement therapy. Moreover, there may be an overlap or transition between constitutional delay in growth and puberty (CDGP) and IHH. It has been increasingly observed that oligogenic inheritance and clinical recovery complicates the phenotype/genotype relationship in IHH, thus making it challenging to find new IHH-associated genes. In a clinical sense, recognizing those IHH genes and associated phenotypes may improve our diagnostic capabilities by enabling us to prioritize the screening of particular gene(s) such as synkinesia (ANOS1), dental agenesis (FGF8/FGFR1) and hearing loss (CHD7). Also, IHH-associated gene studies may be translated into new therapies such as for polycystic ovary syndrome. In a scientific sense, the most significant contribution of IHH-associated gene studies has been the characterization of the long-sought gonadotropin releasing hormone pulse generator. It appears that genetic studies of IHH will continue to advance our knowledge in both the biological and clinical domains.

## INTRODUCTION

The activity level of the hypothalamo-pituitary-gonadal (HPG) axis is remarkably variable throughout life. A gradual increase of HPG activity around the beginning of the second decade of life brings about sex-specific, secondary sexual features and a maturing reproductive system. This specialized phase of human development is called puberty and lasts from two to five years. Absence of puberty manifests itself as sexual immaturity and reproductive incompetence, which can be succinctly termed as hypogonadism. If lack of such development is due to anatomical or functional defects, resulting in reduced gonadotropin releasing hormone (GnRH) and/or gonadotropin release, the condition is called hypogonadotropic hypogonadism (HH).

### 1. Idiopathic Hypogonadotropic Hypogonadism

The term idiopathic HH (IHH) is used to define those IHH cases with no apparent causes. Traditionally, IHH is divided into two major categories: Kallmann syndrome (KS) and normosmic IHH (nIHH). IHH can be congenital or acquired. The great majority of hereditary causes of IHH are congenital. Typically, in girls there is no clinical manifestation of IHH before the early teen years. In boys, since the HPG axis is very active roughly between the 16^th^ and 22^nd^ week of gestation and androgenic end products of this period are required for normal virilization of the 46,XY fetus, male infants with IHH may have micropenis and/or cryptorchidism at birth. Under-virilization of the male can be severe enough to call for an evaluation of a “disorder of sexual development”. A slight and temporary reactivation of the HPG axis in early infancy (around four to sixteen weeks) is called “minipuberty” and provides a unique opportunity to diagnose both male and female infants with congenital IHH (1).

KS is often due to the embryonic maldevelopment and/or interrupted migration of GnRH specific neurons. Since the embryonic migration of GnRH neurons from the nasal placode towards their final destination in the hypothalamus occurs in association with olfactory receptor neurons, the resulting phenotype includes anosmia in addition to HH. KS cases often have additional congenital anomalies such as cleft palate, unilateral renal agenesis, split hands and feet, short metacarpals, deafness, and mirror movements (synkinesia).

In contrast nIHH refers to those IHH cases not associated with anosmia (2). nIHH results from the dysfunction of the normally sited GnRH neurons in the hypothalamus. These cases typically do not have any accompanying congenital lesions.

However, one should be careful when using these terms because the line between KS and nIHH is sometimes blurred, as most typically seen with FGFR1 mutations. Furthermore, there may be pathophysiological overlaps between the two entities. For example, patients with CCDC141 or IGSF10 mutations have nIHH despite showing in vitro evidence of impaired migration of the GnRH neurons (3,4).

Pubertal delay is the most typical presentation of IHH. Pubertal delay is defined as absence of breast development (Tanner breast stage 1) in a girl at age 13 or failure to achieve a testicular volume of 4 mL in a boy by age 14 (5). By far the most common cause of delayed puberty is constitutional delay in growth and puberty (CDGP), which is not a disease per se but a maturational delay in development at the extreme of the population standards. CDPG accounts for pubertal delay in two third of boys and one third of girls (6). CDGP is a diagnosis of exclusion and should often be considered in the differential diagnosis of IHH. To distinguish between these two conditions often requires lengthy workup and observation periods.

It has been shown that some variants in known puberty genes such as TAC3 and TACR3 are shared by individuals with IHH or CDGP within the same family, suggesting that CDGP shares an underlying pathophysiology with IHH, only representing a milder form of the same genetic dysfunction (7). Clinicians often successfully try a low dose sex steroid course to “jump start” pubertal development in patients with suspected CDGP. It is now well established that about 10-20% of IHH cases recover either spontaneously or more typically after receiving some sex steroid replacement therapy (8,9). These foregoing observations further suggest that CDGP and IHH may have common pathophysiological underpinnings. Therefore, it appears that there is a continuum of phenotype from normal timing of pubertal development all the way to extreme IHH, encompassing CDGP along the way.

### 2. Genes Associated with Idiopathic Hypogonadotropic Hypogonadism

Currently known genetic defects account for up to 50% of all IHH cases (10). To date mutations in around 50 genes have been reported to cause IHH. The full current list of genes associated with IHH is shown in Table 1. Presence of more than one IHH-associated mutant gene in a patient/pedigree (oligogenic inheritance) is thought to account for 10-20% of all IHH cases (11,12,13,14). With the increasing use of unbiased comprehensive genetic studies such as whole exome sequencing (WES), it is now known that oligogenic inheritance is more common than previously thought in various Mendelian disorders (15).

### 2a. Kallmann Syndrome Associated Genes

X-linked recessive, autosomal dominant (AD) and autosomal recessive (AR) patterns of inheritance have been reported. However, KS is often sporadic; even if it is familial, a substantial variability in clinical phenotype of the same genetic defect among affected family members may be seen (16,17,18). According to the presence of certain associated clinical features, genetic screening for particular gene(s) may be prioritized: synkinesia (KAL1), dental agenesis (FGF8/FGFR1), digital bony abnormalities (FGF8/FGFR1) and hearing loss (CHD7, SOX10) (19). As a common pathophysiological denominator with KS genes, fibroblast growth factor signaling, prokineticin signaling and Anosmin-1 appear to interact with heparin sulfate glycosominoglycan compounds within an extracellular signaling complex to promote GnRH neuronal migration (20,21).

### ANOS1 (KAL1)

The ANOS1 gene, encoding an extracellular glycoprotein called Anosmin-1, associates with the cell membrane via heparin sulphate proteoglycans (HSPG) (22). Ten to twenty percent of males with KS carry KAL1 mutations or intragenic microdeletions are present (23,24). Most pathogenic mutations entirely disrupt protein function. The inheritance pattern is X-linked recessive. The KS phenotype produced by ANOS1 mutations seem not only more severe but also less variable than that seen with other known molecular defects (24,25). Accompanying clinical features include synkinesia and unilateral renal agenesis, which occurs in 75% and 30% of patients respectively (26).

### FGFR1, FGF8 and Related Genes (FGF17, IL17RD, DUSP6, SPRY4, FLRT3, and KLB) (20,27,28)

FGFR1 requires both HSPG as a co-receptor and Anosmin-1, which is also HSPG-associated. Anosmin-1 is likely to play a role in mediating FGFR1 signaling (21). Loss of FGFR1 function has been reported to elicit reproductive abnormalities ranging from severe AD KS through fully penetrant nIHH to delayed puberty (29,30,31,32,33). Around 10% of patients with KS were found to have inactivating mutations in FGFR1 (20,29,30). More recently, loss-of-function mutations in FGFR1 were detected in 7% of 134 nIHH patients, suggesting that FGFR1 should be one of the major genes in screening panels for nIHH patients (34).

In 2008, FGF8, one of 11 ligands of FGF signaling was found to be mutated in six out of 461 (1.5%) IHH patients. These patients exhibited varying levels of olfactory function and HH (27). Furthermore, mice homozygous for the hypomorphic FGF8 allele exhibited absent olfactory bulbs and lacked GnRH neurons in the hypothalamus (27). As for the features of FGF8/FGFR1 loss of function, cleft palate is found in up to 30% of patients, while cartilage abnormalities in either ear or nose and some digital anomalies have been reported (26). Further screening for FGF8 related genes in a group of 388 congenital IHH patients revealed inactivating variants in FGF17, IL17RD, DUSP6, SPRY4, and FLRT3 (28).

### KLB

KLB is the most recently reported Fibroblast growth factor related IHH gene (35). KLB encodes for Beta-Klotho, which is a co-receptor in FGF21 signaling through the FGFR1 product. The authors of this paper screened more than 300 IHH patients and found 13 patients with loss of function mutations. They also reported that the majority of patients with KLB mutations exhibited some degree of metabolic defect such as insulin resistance or dyslipidemia. The KLB knock out mouse model revealed a milder hypogonadal phenotype when compared to the corresponding human phenotype (35).

### PROKR2 and PROK2

The PROK2 gene encodes prokinetecin 2, an 81 amino acid peptide that signals via the G protein-coupled product of the PROKR2 gene. This ligand and its receptor were recognized as strong candidates for KS as PROK2 (36,37) or PROKR2 knockout mice had defective olfactory bulbs and failed migration of GnRH neurons (38). Subsequently, inactivating variants in PROKR2 or PROK2 were detected in KS patients. Most of these mutations were heterozygous, although both homozygous and compound heterozygous mutations have been described (39). Patients with PROK2 or PROKR2 mutations have considerable phenotypic variability (37,40,41), ranging from KS to nIHH. A variety of accompanying clinical features including fibrous dysplasia, synkinesia and epilepsy have been reported in patients with PROK2 or PROKR2 mutations. It appears that mutations in PROKR2 and PROK2 are often found in combination with other mutations in IHH with oligogenic inheritance.

### CHD7

The CHD7 gene encodes a chromatin-remodeling factor and is mutant in CHARGE syndrome, which has the constellation of Colobomata, Heart Anomalies, choanal Atresia, Retardation, Genital and Ear anomalies (42). Some patients also have IHH and hyposmia. Based on the hypothesis that KS and nIHH may be a milder allelic variant of CHARGE syndrome, CHD7 was screened in 197 patients with KS or nIHH but devoid of CHARGE features. Mutations were identified in three KS and four nIHH patients (43). In another study, three of 56 KS/nIHH patients had mutations in CHD7 (44). The authors suggest that patients diagnosed with KS should be screened for clinical features consistent with CHARGE syndrome. If such features are present, particularly deafness, anomalous ears, coloboma and/or hypoplasia or aplasia of the semicircular canals, CHD7 should be tested (44).

### WDR11

The WDR11 gene product partners EMX1, a homeodomain transcription factor involved in the development of olfactory neurons. By positional cloning, heterozygous mutations were discovered in several patients with KS (45). Recently, a digenic combination of monoallelic variants in PROKR2 and WDR11 has been reported to be responsible for a pituitary stalk interruption syndrome in a child (46).

### SEMA3A

SEMA3A encodes for semaphorin 3A, a protein that interacts with neuropilins. Mice lacking semaphorin 3A expression have been demonstrated to have a Kallmann-like phenotype. Screening large groups of patients with KS revealed a variety of monoallelic mutations. Some of these mutations coexist with other KS causing gene mutations, further showing oligogenic inheritance in IHH (47,48). In a recent study in patients with IHH, heterozygous missense variants in SEMA3A and SEMA7A were found in association with second variants in other IHH genes (49).

### SEMA3E

Semaphorin 3E (SEMA3E) is a secreted protein that modulates axonal growth. A SEMA3E missense mutation was recently reported in two brothers with KS (50). Functional studies have shown that SEMA3E may act as a survival factor for maturing hypothalamic GnRH neurons.

### SOX10

Inactivating mutations in SOX10 cause Waardenburg syndrome, a rare disorder characterized by pigmentation abnormalities and hearing impairment. Screening for SOX10 mutations in KS patients with deafness revealed inactivating variants in approximately one-third of them. SOX10 knockout mice showed absence of olfactory ensheathing cells along the olfactory nerve pathway (51).

### HS6ST1

HS 6-O-sulfotransferase 1 is a sulfation enzyme that specifically and non-randomly modifies heparan sulfate, an important extracellular matrix component, which is probably required for optimal cell-cell communication, such as during olfactory neuronal migration and ligand-receptor interactions. Recently, inactivating HS6ST1 mutations, in association with other KS gene mutations, have been reported in seven families with KS (52).

### CCDC141

CCDC141 encodes a coiled-coil domain containing protein that is expressed in GnRH neurons. We have reported inactivating CCDC141 variants in four separate families with IHH. Affected individuals had normal olfactory function and anatomically normal olfactory bulbs (53). In a rodent nasal explant model, knockdown of CCDC141 resulted in decreased embryonic GnRH cell migration without interrupting olfactory axon outgrowth (3).

### FEZF1

FEZF1 encodes a transcriptional repressor that is expressed during embryogenesis in the olfactory epithelium, amygdala and hypothalamus. The FEZF1 gene product promotes the presence of a protease to enable olfactory receptor neurons, and thus accompanying GnRH neurons, to enter the brain (54). Recently, using autozygosity mapping and WES in a cohort of 30 individuals with KS, we identified homozygous, loss-of-function mutations in FEZF1 in two independent consanguineous families, each with two affected siblings (55).

### IGSF10

GSF10 is a member of the immunoglobulin superfamily. Howard et al (4) obtained WES data on more than 100 individuals with delayed puberty and identified IGSF10 mutations in six families. The knock down studies revealed reduced GnRH migration in the GN11 cell line. Despite having impaired migration of GnRH neurons, the patients carrying these mutations had a normal sense of smell. The authors suggested that reduced number or delayed arrival of neurons in the hypothalamus leads to a somewhat milder functional defect in the formation of the GnRH neuronal network with eventual delayed puberty but not permanent IHH. Interestingly, they also identified mutations in adult individuals with functional hypothalamic amenorrhea, which is considered a form of mild, transient HH (4).

### SMCHD1

SMCHD1 encodes for an epigenetic repressor which is expressed in the human olfactory epithelium. Shaw et al (56) demonstrated inactivating SMCHD1 mutations as the cause of congenital absence of nose in 41 cases. The great majority of patients (97%) also had hypogonadal features such as cryptorchidism, microphallus or amenorrhea, along with absent olfactory structures and anosmia.

### 2b. Normosmic Idiopathic Hypogonadotropic Hypogonadism (nIHH) Associated Genes

nIHH-causing genes are more pertinent to the understanding of the function of the HPG axis and puberty. Identified mutations in familial cases of nIHH has led to greater understanding of this function. In a study on 22 consecutive, multiplex families with nIHH, we identified mutations in five genes (GNRHR, TACR3, TAC3, KISS1R, and KISS1) in 77% of them. GNRHR and TACR3 mutations were the two most common causative mutations, occurring with about equal frequency in two third of the mutation identified cases (57).

### LEP and LEPR

Leptin deficiency with mutations in either encoding leptin (LEP) or encoding the leptin receptor (LEPR) is associated with IHH (58,59). The administration of leptin in LEP-deficient patients restores normal pubertal development but does not cause early puberty in prepubertal children, which implies that leptin is a permissive factor for the development of puberty in humans (60). These patients are easily recognizable among other IHH patients with because of the presence of early onset obesity and hyperphagia.

### NR0B1 (DAX1)

NR0B1 is an orphan member of the nuclear receptor superfamily. Inactivating variants in the NR0B1 gene cause X-linked congenital adrenal hypoplasia with HH (61). Adrenal hypoplasia typically presents as adrenal insufficiency during infancy, whereas HH becomes manifest in affected males who survive into the second decade of life.

### SRA1

SRA1 was the first gene shown to function through both its protein and noncoding, functional RNA products (62). These products act as co-regulators of nuclear receptors, including sex steroid receptors as well as SF-1 and LRH-1, the master regulators of steroidogenesis. SRA1 is required for the synergistic enhancement of SF-1 transcriptional activity by DAX-1 (NR0B1), mutations in which also cause IHH, as discussed above (63). WES and autozygosity mapping studies revealed three independent families in which IHH was associated with inactivating SRA1 variants (64).

### GNRHR and GNRH1

GNRH1 and GNRHR are the most obvious candidate gene in the etiology of IHH. GNRHR defects produce AR, isolated nIHH, with no evidence of accompanying developmental defects such as hyposmia (65,66,67). GNRHR mutations have been suggested to account for about 40-50% of familial AR nIHH, and around 17% of sporadic nIHH (66). In a recent survey of 110 patients with nIHH, eleven IHH patients (10%) carried biallelic GNRHR mutations while none of the 50 patients studied with CDGP had any deleterious variants (68). To date, more than 25 different mutations have been reported. Interestingly, only seven years ago the first inactivating homozygous mutations in GNRH1 itself causing IHH were reported by two independent groups (69,70). In these cases IHH was shown to be reverseable by pulsatile GnRH administration, confirming the pivotal role of GnRH in human reproduction (69). Out of 310 patients with IHH, only one case was found, attesting to the rarity of mutations in this gene as a cause of IHH (70). We recently reported further GNRH1 mutations located in the region encoding the decapeptide which is the same region involved in earlier reported mutations (71).

### KISS1R and KISS1

KISS1R (formerly GPR54) encodes for the receptor for small peptides derived from the KISS1 gene and it was previously thought not to play a role in the HPG axis (72). Mutations in KISS1R were first reported in IHH familial multiplex cases in 2003 (73,74). Ensuing studies established kisspeptin signaling as an essential, positive regulator of GNRH secretion. In a mutational screening study, only five out of 166 (3%) probands with nIHH were found to have rare variants in KISS1R (75). Studying a large, consanguineous family with four sisters with nIHH, we found inactivating mutations altering the 4th amino acid of Kisspeptin-10. Overnight frequent LH sampling did not reveal any LH pulsatility, further confirming the essential role of kisspeptin signaling in the GnRH pulse generator (76).

### TACR3 and TAC3

Tachykinin receptor-3 encoded by TACR3 is the mediator of biologic actions of neurokinin B (NKB) encoded by TAC3. In an effort to identify novel genes playing a role in driving the HPG axis, based on autozygosity mapping (77), we identified homozygous non-synonymous mutations in the coding sequences of TAC3 or TACR3 in nine patients from four families with an nIHH phenotype (78). With the additional cases identified in our cohort, it became clear that TACR3 mutations are almost as common as GNRHR mutations (57). Other groups have made similar observations concerning the prevalence of TACR3 mutations. Gianetti et al (79) found 19 among 345 (5.5%) cases while a very similar rate (5.2%) was observed by Francou et al (80) from a cohort of 173 cases of familial and sporadic nIHH. The frequent presence of a micropenis and cryptorchidism in mutant TACR3 male patients indicates that intact TACR3 function is also required for normal fetal gonadotropin secretion, which stimulates testicular size and descent and penile growth (1).

Clinical reversibility, evident by spontaneous progression of puberty, often following a period of exogenous sex steroid treatment, was seen in 10% of an unselected nIHH cohort (8). A much greater percentage of reversibility (83%) was reported by Gianetti et al (79) in their TAC3/TACR3 cohort 2010 (79). In our cohort four patients from three independent and ethnically different families showed clinical recovery among 16 (25%) patients. Interestingly, all of these families harbored the same TACR3 mutation (p.T177K). Our studies are ongoing in an attempt to gain insight into the clinical recoverability and/or reversibility of this variant. With such a high rate of reversibility, a legitimate question arose as to whether CDGP was a form of IHH caused by TACR3 mutations. To answer this question, Vaaralahti et al (81) screened these genes in 146 Finnish subjects with CDGP and found no variants to account for this phenotype.

Other clinical studies have provided additional valuable insight in to the biology of the HPG axis. Young et al (82) were able to produce pubertal levels of gonadotropin and sex steroids with repeated administration of GnRH in patients with Null mutations in TAC3, indicating that the site of NKB action is proximal to GnRH and the pituitary (82).

### 3. Scientific Significance of Identifying IHH-Associated Genes

Undoubtedly, the most significant contribution of IHH-associated gene studies has been the characterization of the long sought-after GnRH pulse generator. A surge of studies over the past ten years on Kisspeptin and NKB signaling, following the identifications of their inactivating mutations among familial patients with nIHH, has led to characterization of the GnRH pulse generator. According to the current understanding there is a network of sex-steroid responsive neurons in the arcuate (infindubular) nucleus that coexpress Kisspeptin, NKB, Dynorphin and ERα (KNDy or Kisspeptin neurons). Within these cells, the stimulatory NKB starts an action potential that is suppressed by the inhibitory Dynorphin. When the inhibitory effect of Dynorphin is overcome another stimulatory NKB action takes over. The net result is continuous, intermittent action potentials. Each action potential translates into a pulsatile secretion of Kisspeptin on to the axons of the GnRH neurons in the median eminence, thence GnRH is released towards the pituitary gonadotropes, via the portal circulation. Synchronization of KNDy cells is believed to be provided by NKB-NK3R signaling through ipsi- and contralateral projections among these cells (83,84,85).

### 4. Clinical Significance of Identifying IHH-Associated Genes

IHH-associated gene studies have provided clues for targetting diagnostic molecular genetic studies. GNRHR and TACR3 should be the first two genes to be screened for diagnostic purposes in a clinical setting for equivocal cases, such as constitutional delay in puberty vs. IHH. In KS, according to the presence of certain accompanying clinical features, genetic screening for particular gene(s) may be prioritized, for example if the patient has synkinesia then KAL1 would be suggested, dental agenesis is associated with FGF8/FGFR1, digital bony abnormalities also with FGF8/FGFR1 and hearing loss with CHD7 and SOX10.

IHH-associated gene studies may be translated into new therapeutic modalities. For instance, an antagonist of the TACR3 gene product has been in clinical trial for polycystic ovarian syndrome (86).

### 5. Concluding Remarks

Currently, around half of the IHH genes remain to be identified. Complicated genotype/phenotype relationships in IHH, due to two well-established phenomena, oligogenic inheritance and spontaneous or induced clinical reversibility, make identifying these unknown genes challenging. Nonetheless, with the help of contemporary sequencing technologies, it appears that studies into the genetics of hypogonadotropic hypogonadism will continue to advance our knowledge in both the biological and clinical domains.

## Figures and Tables

**Table 1:**
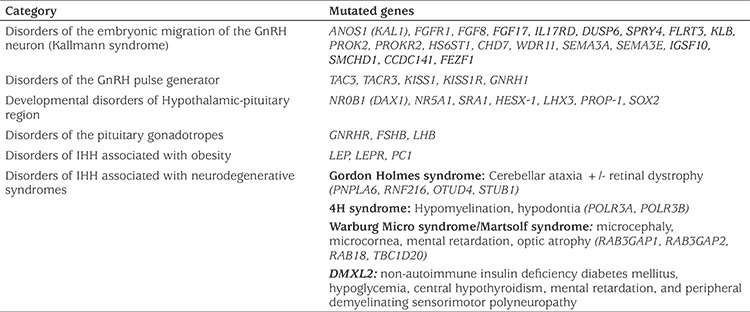
Genetic causes of idiopathic hypogonadotropic hypogonadism
